# Direct whole-genome sequencing of *Coxiella burnetii* from environmental samples using a two-step selective enrichment approach

**DOI:** 10.1371/journal.pntd.0014716

**Published:** 2026-09-08

**Authors:** Mikhail Bezruchko, Olga Freylikhman, Erik Khalilov, Elena Vakalova, Denis Kasatkin, Sofia Platova, Ivan Gorokhov, Alina Saitova, Islam Karmokov, Nikolay Tokarevich, Dmitrii Polev, Vladimir Dedkov

**Affiliations:** 1 Saint-Petersburg Pasteur Institute, St. Petersburg, Russia; 2 North-West Anti-Plague Station, St. Petersburg, Russia; 3 Center of Hygiene and Epidemiology in Astrakhan region, Astrakhan, Russia; 4 Martsinovsky Institute of Medical Parasitology, Tropical and Vector Borne Diseases, Sechenov First Moscow State Medical University, Moscow, Russia; Colorado State University, UNITED STATES OF AMERICA

## Abstract

Current high-throughput sequencing platforms enable routine whole-genome sequencing of bacteria via clonal amplification from primary samples, followed by DNA extraction, library preparation, and sequencing. This approach enriches bacterial DNA while removing contaminating nucleic acids, allowing efficient genomic sequencing using minimal sequencing resources per sample. However, for fastidious or obligate intracellular bacteria, such as *Coxiella burnetii*, cultivation is often impractical, leaving pathogen DNA heavily diluted by host and environmental nucleic acids. In such cases, sequencing requires substantially greater throughput per sample, highlighting the need for culture-independent methods to support genomic epidemiology and surveillance. Here, we report a novel two-stage DNA enrichment strategy applied to *C. burnetii*-contaminated sheep wool samples. Such samples feature very low pathogen biomass and a high host-DNA background. Our approach integrates (i) selective whole-genome amplification of complex DNA extracts, followed by (ii) in-solution hybridization capture using custom double-stranded DNA baits. Critically, we developed a WGA-based method to synthesize whole-genome dsDNA baits from minimal input DNA, enabling efficient bait production even in the absence of cultured isolates. Notably, we demonstrate that baits derived from *C. burnetii* DNA contaminated with an unrelated host (e.g., chicken) remain effective for enriching *C. burnetii* from samples derived from a different host species (e.g., sheep), indicating robust cross-host applicability. Using this workflow, we achieved up to 95% *C. burnetii* enrichment and generated six genomic assemblies suitable for downstream phylogenomic and epidemiological analyses. This cultivation-free, two-stage enrichment framework can be applied for phylogenetics and epidemiology and may significantly expand the feasibility of genomic studies for intracellular and otherwise uncultivable bacterial pathogens.

## Author summary

While detecting specific bacterial pathogens is straightforward using standard PCR, understanding their evolution and spread requires whole-genome sequencing. However, for hard-to-culture pathogens like *Coxiella burnetii*, obtaining pure bacterial DNA for sequencing is highly challenging. These bacteria grow inside host cells, resulting in low bacterial DNA yields and high contamination from the host. Consequently, their genomic diversity remains poorly understood. To overcome this, we developed a two-step DNA enrichment method combining selective whole-genome amplification with hybrid capture. This approach enables the sequencing of *Coxiella* genomes directly from complex biological or environmental samples, bypassing the need for prior bacterial culturing. It efficiently enriches target DNA even when total yields are low and host contamination is high. Additionally, we pioneered the use of Phi29 polymerase to generate hybridization probes, allowing rapid production of abundant probes from minimal starting material. Crucially, we demonstrated that these probes can be synthesized from mixed DNA preparations. For instance, DNA from *Coxiella* cultures grown in chicken cells can be used to capture *Coxiella* DNA from mammalian or environmental samples, provided the host species are evolutionarily distant. By eliminating the culturing bottleneck, our method significantly expands the capacity for genomic surveillance and epidemiological studies of difficult-to-cultivate pathogens.

## Introduction

*Coxiella burnetii*, the causative agent of Q fever, is a pathogen of significant public health concern due to its unique biological properties. These include an obligate intracellular life cycle, environmental persistence, diverse spectrum of animal reservoirs, and low infectious dose. The aforementioned features also render it a potential bioterrorism agent [1]. These characteristics contribute to its ability to cause both sporadic cases and large-scale outbreaks, a trend supported by documented increases in global incidence [2–7].

Despite its epidemiological importance, Q fever remains substantially underdiagnosed, and its true prevalence and the distribution of its natural reservoirs are poorly characterized [5–17]. This diagnostic and surveillance gap highlights the critical need for robust molecular tools. Genotyping is essential for outbreak investigation, source tracking, and understanding transmission dynamics [8,12–22]. However, the successful application of genotyping methods, from classical techniques like multispacer sequence typing (MST) and multiple-locus variable-number tandem repeat analysis (MLVA) [9,10,23–28] to advanced whole-genome sequencing (WGS), is often hindered by the low concentration of *C. burnetii* DNA in clinical and environmental samples. This limitation stems from low bacterial loads, the pathogen’s intracellular nature, and high levels of host DNA contamination.

Whole-genome sequencing offers unparalleled resolution for the genomic epidemiology of pathogens, such as *C. burnetii*, enabling detailed analysis of virulence, evolution, and transmission [21,24–26,29]. However, its application to native samples is frequently precluded by an insufficient amount of target DNA, as conventional culture-based amplification is often impractical or impossible [30]. While *in vitro* DNA enrichment methods exist, there remains a clear need for more robust, cost-effective, and universally applicable protocols to generate high-quality WGS data from challenging, low-input samples.

To address this, we developed a two-stage enrichment protocol combining selective whole-genome amplification [28] with a hybridization capture step using cost-effective (self-made) mixed-species DNA probes.

As a proof-of-principle, we applied the protocol to six ovine wool samples naturally contaminated with *C. burnetii*. These samples were specifically selected based on their low qPCR Cq values to demonstrate the method’s efficacy. We evaluated its performance across several key parameters: (i) the proportion of reads mapping to *C. burnetii*, (ii) genomic coverage at 1 × , 10 × , 20 × , and 30 × depth thresholds, (iii) assembly completeness based on core gene sets, and (iv) data quality sufficient for downstream SNP analysis.

Finally, to determine the operational limits of the assay in its current implementation, we evaluated an artificial dilution series of *C. burnetii* DNA mixed with human DNA. This allowed us to define the specific range of target DNA concentrations, as determined by qPCR Cq values, at which a sufficient proportion of on-target reads can be obtained to achieve a near-complete genome assembly. Collectively, this study provides a robust solution for sequencing hard-to-culture pathogens from samples with low DNA yield, enhancing genomic surveillance capabilities.

## Methods

### Ethics statement

In accordance with the law and institutional requirements, no ethics approval was required for research involving animals insofar as samples were obtained as part of official anti-epidemic measures.

### Sampling

The study utilized DNA samples extracted from sheep wool collected from livestock farms where *C. burnetii* DNA had previously been detected during routine Q fever surveillance screening as part of farm health monitoring programs. Wool specimens were individually collected from six sheep (N = 6) residing on private backyard farms in Astrakhan Oblast, Russia, between May and September 2024. A total of six 10-gram wool samples were obtained, with wool taken from five anatomical sites per animal, including visibly soiled regions of the fleece. Samples were stored in sterile, dry 50 mL polypropylene tubes and transported to the laboratory within 24 hours under refrigerated conditions (2–8°C) for further processing. Sample preparation involved mincing wool tufts with scissors, followed by the addition of 1 × PBS buffer at a 1:5 (w/v) ratio, specifically, 1 part wool by weight to 5 parts buffer by volume. This was followed by vigorous shaking of the sealed tube for 5 minutes. The resulting aqueous fraction was then used for DNA extraction.

### DNA extraction and PCR screening

DNA extraction was performed from 100 µL of the wool wash solution using the RIBO-prep RNA/DNA extraction kit (Central Research Institute of Epidemiology, Rospotrebnadzor, Russia) according to the manufacturer’s instructions. The purified DNA was eluted in 50 µL of elution buffer and stored at –20 °C until further use.

Detection of *C. burnetii* DNA in the studied samples was carried out by real-time PCR (qPCR) using the specific *Coxiella burnetii*-FL kit (AmpliSens, Russia) following the manufacturer’s protocol. Each 25 µL reaction contained 10 µL of extracted DNA. Amplification was performed on a CFX96 C1000 Real-Time PCR Detection System (Bio-Rad, USA). Following the manufacturer’s instructions, samples with a threshold cycle value of ≤40 were considered positive. For the present study, we included all available DNA extracts from our laboratory repository that met a stricter inclusion criterion of a Cq value <20. A total of six samples satisfied this requirement, with Ct values ranging from 14 to 16.

### DNA enrichment by selective whole genome amplification using Phi29 polymerase

We performed selective whole-genome amplification (SWGA) to enrich target DNA (*C. burnetii*) using a Phi29 polymerase-based method as reported by Cocking *et al*. (2020) [28]. All primers from the published set were custom-synthesized by Oligomatrix (St. Petersburg, Russia) and subsequently pooled to a working concentration of 100 µM, equivalent to 2.5 µM per individual primer. The original amplification protocol was modified as follows. To 4 µL of each DNA sample, we added 2 µL of 10 × Phi29 DNA Polymerase Buffer (TransGen Biotech Co., Ltd.), 4 µL of the primer pool (yielding a final concentration of 0.5 µM per primer), 1 µL of 25 mM dNTPs, 8 µL of nuclease-free water (ddH_2_O), and 1 µL (250 U) of Phi29 DNA polymerase (TransGen Biotech Co., Ltd.), resulting in a total reaction volume of 20 µL. Amplification was performed using a stepwise temperature program: 35°C for 5 min, 34°C for 10 min, 33°C for 15 min, 32°C for 20 min, 31°C for 30 min, followed by 30°C for 12 h. This was followed by enzyme inactivation (65°C, 15 min) and a subsequent hold (4°C). Following amplification, 130 µL of ddH_2_O was added to each reaction, and DNA concentration was quantified using a Qubit 4.0 fluorometer (Thermo Scientific, USA) with Pico488 dye (Lumiprobe, Russia).

The amplified DNA products were used to prepare libraries for next-generation sequencing (NGS). Library preparation was performed using the NadPrep EZ DNA Library Preparation Kit v2 (Nanodigmbio, China), to achieve a target insert size of approximately 300 bp. Sequencing was carried out on the DNBSEQ-G50 platform (MGI, China) in paired-end mode with a read length of 150 nucleotides.

Following sequencing, the proportion of *C. burnetii* genomic DNA in the selectively amplified samples was assessed. Specifically, the fraction of *C. burnetii* DNA was estimated as the percentage of sequencing reads that aligned to the *C. burnetii* reference genome using the BWA-MEM algorithm with a minimum seed length parameter (-k) set to 80.

### Preparation of hybridization probes

Hybridization probes were generated from genomic DNA of the *C. burnetii* vaccine strain M44, originally propagated in chicken embryos. To produce the probes, the DNA was first amplified using Phi29 DNA polymerase in the presence of biotinylated nucleotides, followed by fragmentation and end repair as detailed below.

Amplification was performed in a total reaction volume of 20 µL using the Phi29 DNA Polymerase Kit (TransGen Biotech Co., Ltd.). The reaction mixture contained: 30 ng of genomic DNA, 2 µL of 10 × Phi29 DNA Polymerase Buffer, 4 µL of Phi29 Random Primers, 1 µL of 25 mM dNTPs, 1 µL of 1 mM biotin-15-dCTP, and 1 µL (250 U) of Phi29 DNA Polymerase. The amplification protocol employed a stepwise temperature program: 35°C for 5 min, 34°C for 10 min, 33°C for 15 min, 32°C for 20 min, 31°C for 30 min, and 30°C for 12 h. This was followed by enzyme inactivation (65°C, 15 min) and a subsequent hold (4°C). After amplification, 130 µL of nuclease-free water (ddH_2_O) was added to each reaction, and DNA concentration was quantified using a Qubit 4.0 fluorometer (Thermo Scientific, USA) with Pico488 dye (Lumiprobe, Russia).

The amplified, biotinylated DNA was then fragmented and subjected to end repair using the NadPrep EZ DNA Library Preparation Kit v2 (Nanodigmbio, China). The resulting probes had a target fragment size of approximately 300 bp. The resulting fragmented and biotinylated DNA served as hybridization probes for in-solution hybrid capture. In parallel, an aliquot of this probe preparation was further converted into a sequencing library using the same NadPrep EZ DNA Library Preparation Kit v2 and sequenced on the DNBSEQ-G50 platform (MGI, China) in paired-end mode (2 × 150 bp) to evaluate probe composition.

### Targeted DNA enrichment by in-solution hybridization

Sequencing libraries were prepared from 20 ng of SWGA-enriched DNA using the NadPrep EZ DNA Library Preparation Kit v2 (Nanodigmbio, China), following the manufacturer’s protocol. Indexed adapters from the TruSeq DNA CD Indexes set (Illumina, USA) were used for the ligation step, followed by final library amplification via 12-cycle PCR with standard Illumina TruSeq PCR Primer Cocktail (PPC).

Individual libraries were pooled to a total DNA amount of 1000 ng, with normalization based on the proportion of *C. burnetii* genomic DNA in each sample, as determined from prior sequencing data. To the pooled library solution, we added 200 ng of the biotinylated hybridization probes generated earlier, 10 µL of COT Human DNA (Roche Diagnostics, Germany), and a double volume of AMPure XP beads (Beckman Coulter). Following a 10 minute incubation at room temperature, magnetic particles were captured using a magnetic rack, washed twice with 80% ethanol, and air-dried for 2 minutes at room temperature. They were then eluted in a mixture containing reagents supplied with the KAPA HyperCapture Reagent Kit (Roche, Germany): 6 µL of Universal Enhancing Oligos, 14 µL of hybridization buffer, 6 µL of hybridization component H, and 3.5 µL of PCR-grade H_2_O. Supernatant containing the eluted DNA was transferred to a new tube, free of magnetic beads.

Hybridization and subsequent wash steps were carried out according to the KAPA HyperCap Workflow v3.2 (Roche, Germany). Post-capture amplification was performed using 20 PCR cycles. Enriched libraries were then sequenced on the MiSeq System (Illumina, USA) in paired-end mode (2 × 250 bp).

### Evaluation of enrichment performance using dilution series

To systematically evaluate the performance of the two-stage enrichment protocol across a range of pathogen DNA concentrations, we prepared a dilution series of *C. burnetii* genomic DNA in a constant background of human genomic DNA (cat. no. NA101, Evrogen, Russia) (20 ng per reaction). The estimated *C. burnetii* input ranged from 2.5 ng down to 0.000032 ng via 5-fold serial dilutions, corresponding to approximately 1.1 × 10⁶ to 1.4 × 10¹ genome equivalents, respectively, based on a *C. burnetii* genome size of ~2.1 Mbp. To verify the initial DNA input, an aliquot of each dilution was tested by qPCR using the *Coxiella burnetii*-FL kit (AmpliSens, Russia) prior to downstream processing. Three independent replicate series were prepared, yielding a total of 24 samples (8 dilutions × 3 replicates).

Each sample was processed under three conditions in parallel: (i) direct sequencing without enrichment (native DNA); (ii) SWGA followed by sequencing; and (iii) SWGA followed by hybridization capture and sequencing. All libraries were independently sequenced on the MiSeq System (Illumina, USA) in paired-end mode (2 × 250 bp).

### Data analysis and visualisation

Raw paired-end (PE) reads were initially analyzed using FastQC software (v0.12.1, Babraham Institute, Cambridge, UK) to assess quality and suitability for further analysis. The reads were trimmed to remove adapters and low-quality sequences (Q-score < 20) and filtered using Trim Galore! (v0.6.7). The parameter ‘-l 100’ was applied to discard read pairs where at least one read was shorter than 100 nucleotides.

Bacterial genomes were assembled *de novo* with SPAdes v4.2.0. The assemblies were filtered to remove contigs shorter than 200 bp and subsequently evaluated for quality using QUAST (v5.2.0) and CheckM (v1.2.4). The final genomic assemblies are available in the CNCB database [31,32] under BioProject PRJCA050322.

Sequencing reads were aligned to the *C. burnetii* RSA 493 reference genome (GCA_000007765.2) using BWA-MEM (v0.7.17-r1188) with the parameters ‘-k 80’ (minimum seed length) and ‘-c 3’ (discard chains with fewer than 3 seeds). The resulting SAM files were processed using SAMtools (v1.19.2) to calculate the proportion of reads that uniquely mapped to the *C. burnetii* genome in each sample. To identify and quantify PCR duplicates, the aligned BAM files were processed using the SAMtools markdup module. Reads were sorted by query name using samtools sort -n, followed by samtools fixmate -m to add mate score (ms) tags, and re-sorted by coordinate. Duplicates were marked without removal using samtools markdup -s. The number of duplicated reads was extracted from the samtools stats output. Coverage statistics at 1 × , 10 × , 20 × , and 30 × thresholds were calculated using mosdepth (version 0.3.10), with PCR duplicates excluded.

For saturation curve analysis, sequencing reads were subsampled in increments of 500,000 reads using seqtk (v1.4-r122) with a fixed seed value (-s 100) to ensure reproducibility. Coverage depth was then calculated for all samples using mosdepth (v0.3.10) based on the alignment of reads to the *C. burnetii* reference genome, utilizing only reads that successfully mapped to the *C. burnetii* genome from the resulting BAM files.

Single nucleotide polymorphism (SNP) calling was performed using Snippy (v1.19.2) with *C. burnetii* RSA 493 (GCF_000007765.2) as reference. Core genome alignment was generated using snippy-core (v4.0.2) and cleaned with snippy-clean_full_aln (v4.0.2). Pairwise SNP distances were calculated with snp-dists (v0.8.2). The phylogenetic tree was constructed using IQ-TREE v3.0.1 under the GTR + G model with 1000 ultrafast bootstrap replicates, rooted with the Coxiella-like endosymbiont strain CRt (CP011126.1) as outgroup. All *Coxiella burnetii* genomes available in the NCBI GenBank database as of November 2025 were included in this study. A complete list of these genomes is provided in S1 Table, which details the assembly accession numbers and a composite identifier incorporating key metadata: assembly level, strain name, plasmid designation, host species, collection date, geographic origin, isolation source, and sample type.

For integrated visualization of the bacterial chromosome, a circular diagram was constructed using Circos (v0.69-9). It simultaneously displays GC content, sequencing coverage depth, and excluded regions for all samples. Coverage was calculated in 250-nucleotide windows using mosdepth (v0.3.10). The visualization is based on normalized sequencing data (2 million reads per sample) that were aligned to a repeat-masked reference genome using BWA-MEM (v0.7.17-r1188) with the parameters ‘-k 80 -c 3’. Repeat masking was performed based on bacterial genome annotation (GTF file), specifically excluding transposons, transposon-associated proteins, and elongation factors.

**Fig 1:**
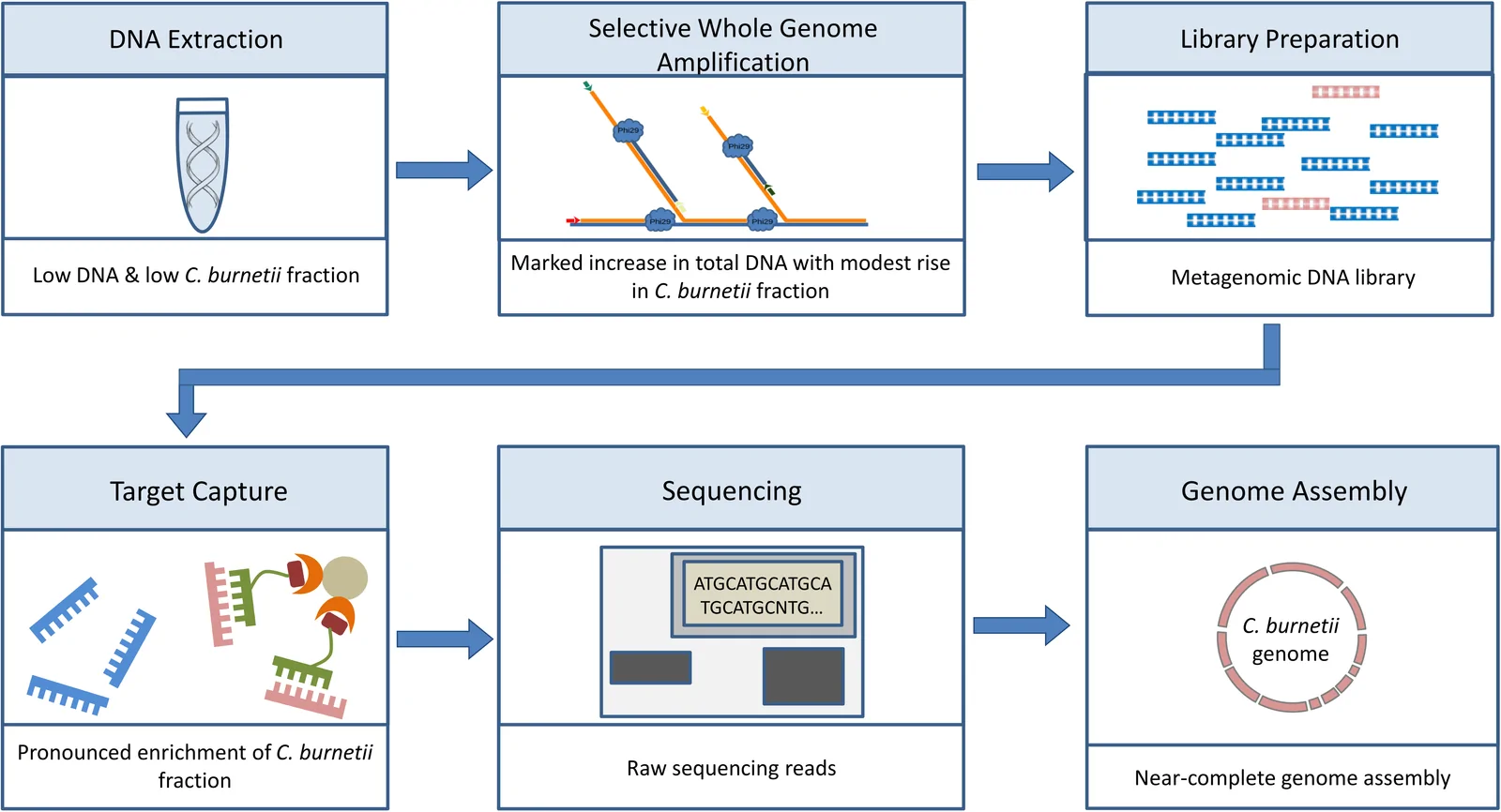
Schematic of *Coxiella burnetii* DNA enrichment workflow.

The schematic representation of the DNA enrichment principle is illustrated in Fig 1, which was created using PowerPoint.

## Results

### Hybridization probe synthesis

Hybridization probes were generated from genomic DNA isolated from *C. burnetii* strain M44 cultured in chicken embryos. These probes consisted of biotinylated, fragmented dsDNA with a median fragment size of approximately 300 bp. To assess probe composition, they were subjected to next-generation sequencing. Subsequent bioinformatic analysis revealed that: at least 46% of the sequenced reads corresponded to *C. burnetii* genomic DNA; approximately 35% aligned to chicken genome (*Gallus gallus*, GCA_016699485.1); and the remaining ~19% showed no significant similarity to the reference genomes used in the analysis (Table 1).

**Table 1:** Hybridization probe composition.

Sample	Total reads. n	*G. gallus* mapped reads, n (%)	*C. burnetii* mapped reads, n (%)	Other reads, n (%)
ZondCox	6600580	2291502 (34.72)	3053523 (46.27)	1255555 (19.01)

### Assessment of *C. burnetii* DNA enrichment via serial dilutions

To evaluate the performance of our enrichment approach across a range of pathogen DNA concentrations, we prepared a 5-fold serial dilution series of *C. burnetii* genomic DNA in a constant background of human genomic DNA (20 ng per reaction). Each dilution was assessed by qPCR prior to enrichment (Table 2), providing a quantitative reference for the initial *C. burnetii* DNA load. These reference points establish a baseline correlation between qPCR Cq values and actual pathogen DNA concentration. Consequently, they allow for the prediction of sequencing outcomes in future samples and help define the practical limit of the assay: specifically, a Cq threshold below which the protocol reliably recovers sufficient data for downstream analysis, and above which the target DNA concentration is likely too low for successful genome reconstruction.

**Table 2:** qPCR assessment of *C. burnetii* DNA across 5-fold dilution series.

Dilution №	*C. burnetii* DNA (ng) in 20 ng human DNA background	Estimated *C. burnetii* genome copies	Cq value (mean ± SD)
0	2.5	~1,140,000	14.27 ± 1.46
1	0.5	~228,500	13.80 ± 0.70
2	0.1	~45,700	16.03 ± 0.06
3	0.02	~9,140	18.20 ± 0.26
4	0.004	~1,828	20.73 ± 0.06
5	0.0008	~366	23.20 ± 0.10
6	0.00016	~73	25.73 ± 0.23
7	0.000032	~15	28.10 ± 0.17

Direct sequencing of non-enriched DNA (Table 3) yielded very low *C. burnetii* read fractions across all dilutions, with the highest value (0.72 ± 0.10%) observed at the initial concentration (dilution 0). Genomic coverage at 1 × reached only 58.0 ± 5.0% at this dilution and declined sharply at subsequent dilutions.

**Table 3:** Sequencing metrics of non-enriched *C. burnetii* DNA across 5-fold dilution series.

Dilution №	*C. burnetii* mapped reads, % (mean ± SD)	*C. burnetii* duplicated reads, % (mean ± SD)	1x coverage, % (mean ± SD)	10x coverage, % (mean ± SD)	20x coverage, % (mean ± SD)	30x coverage, % (mean ± SD)
0	0.72 ± 0.10	0.071 ± 0.009	58.00 ± 5.00	0 ± 0	0 ± 0	0 ± 0
1	0.068 ± 0.023	0.047 ± 0.081	8.30 ± 2.30	0 ± 0	0 ± 0	0 ± 0
2	0.0127 ± 0.0064	0 ± 0	1.30 ± 0.60	0 ± 0	0 ± 0	0 ± 0
3	0.00294 ± 0.00113	0 ± 0	0 ± 0	0 ± 0	0 ± 0	0 ± 0
4	0.000241 ± 0.000078	0 ± 0	0 ± 0	0 ± 0	0 ± 0	0 ± 0
5	0.000125 ± 0.000014	0 ± 0	0 ± 0	0 ± 0	0 ± 0	0 ± 0
6	0.000036 ± 0.000062	0 ± 0	0 ± 0	0 ± 0	0 ± 0	0 ± 0
7	0 ± 0	0 ± 0	0 ± 0	0 ± 0	0 ± 0	0 ± 0

SWGA amplification alone (Table 4) provided moderate improvement, increasing the *C. burnetii* read fraction to 2.63 ± 0.19% at dilution 0, with 1 × coverage reaching 88.0 ± 1.0%. However, the enrichment effect diminished rapidly at lower DNA inputs. While SWGA enhanced the recovery of *C. burnetii* reads compared to native DNA, it remained insufficient for recovering complete genomic data across the dilution series.

**Table 4:** Sequencing metrics and genomic coverage after SWGA enrichment across 5-fold dilution series.

Dilution №	*C. burnetii* mapped reads, % (mean ± SD)	*C. burnetii* duplicated reads, % (mean ± SD)	1x coverage, % (mean ± SD)	10x coverage, % (mean ± SD)	20x coverage, % (mean ± SD)	30x coverage, % (mean ± SD)
0	2.63 ± 0.19	0.104 ± 0.006	88.0 ± 1.0	2.7 ± 0.6	0 ± 0	0 ± 0
1	0.53 ± 0.07	0.089 ± 0.011	44.0 ± 3.0	0 ± 0	0 ± 0	0 ± 0
2	0.100 ± 0.017	0.020 ± 0.035	10.7 ± 1.5	0 ± 0	0 ± 0	0 ± 0
3	0.0156 ± 0.0017	0 ± 0	1.7 ± 0.6	0 ± 0	0 ± 0	0 ± 0
4	0.00260 ± 0.00023	0 ± 0	0 ± 0	0 ± 0	0 ± 0	0 ± 0
5	0.00050 ± 0.00020	0 ± 0	0 ± 0	0 ± 0	0 ± 0	0 ± 0
6	0.000022 ± 0.000037	0 ± 0	0 ± 0	0 ± 0	0 ± 0	0 ± 0
7	0 ± 0	0 ± 0	0 ± 0	0 ± 0	0 ± 0	0 ± 0

In contrast, the two-stage enrichment approach with SWGA followed by hybridization capture dramatically improved sequencing efficiency (Table 5). At dilutions 0–2, *C. burnetii* read fractions exceeded 87%, with near-complete 1 × coverage (99%) and substantial coverage at higher depth thresholds (20× and 30×). At dilution 3, which corresponded to a Cq value of approximately 18.20 (Table 2), the *C. burnetii* read fraction remained high (63.64 ± 1.32%), with 30 × coverage reaching 53.33 ± 4.16%. However, at dilution 4 (Cq ≈ 20.73), performance declined markedly, with the *C. burnetii* read fraction dropping to 13.57 ± 0.78%. Notably, even at the lowest DNA inputs (dilutions 6–7, Cq ≈ 25.73–28.10), where no *C. burnetii* reads were detected in non-enriched or SWGA-only libraries, hybridization capture enabled the recovery of *C. burnetii* reads (0.31 ± 0.17% and 0.02 ± 0.01%, respectively). PCR duplication rates increased as the *C. burnetii* DNA input decreased, rising from 2.20 ± 0.26% at dilution 0 to 66.86 ± 3.55% at dilution 6. In terms of sequencing output, on the order of 600,000 individual reads mapped to the reference genome provided sufficient data for 99% 1 × genome coverage and >90% 10 × coverage. This read count was achieved at dilution 3 with approximately 945,000 raw reads for sample S3-3AZ (see Tab AZ in S2 Table).

**Table 5:** Sequencing metrics and genomic coverage after SWGA and hybridization capture enrichment across 5-fold dilution series.

Dilution №	*C. burnetii* mapped reads, % (mean ± SD)	*C. burnetii* duplicated reads, % (mean ± SD)	1x coverage, % (mean ± SD)	10x coverage, % (mean ± SD)	20x coverage, % (mean ± SD)	30x coverage, % (mean ± SD)
0	94.88 ± 0.82	2.20 ± 0.26	99 ± 0	98 ± 0	97 ± 0	93.67 ± 0.58
1	93.15 ± 1.11	2.68 ± 0.58	99 ± 0	98.33 ± 0.58	97.33 ± 0.58	94.33 ± 1.53
2	87.73 ± 1.76	12.00 ± 0.55	99 ± 0	93.33 ± 0.58	76.33 ± 1.53	58.33 ± 2.31
3	63.64 ± 1.32	13.76 ± 0.41	99 ± 0	91.33 ± 1.53	71.67 ± 4.04	53.33 ± 4.16
4	13.57 ± 0.78	51.56 ± 1.35	64.33 ± 0.58	3 ± 0	1 ± 0	1 ± 0
5	3.00 ± 1.01	50.55 ± 1.44	49.33 ± 4.04	4.33 ± 0.58	1.67 ± 0.58	1 ± 0
6	0.31 ± 0.17	66.86 ± 3.55	5 ± 1	0 ± 0	0 ± 0	0 ± 0
7	0.02 ± 0.01	52.64 ± 5.13	2.33 ± 0.58	0 ± 0	0 ± 0	0 ± 0

**Fig 2:**
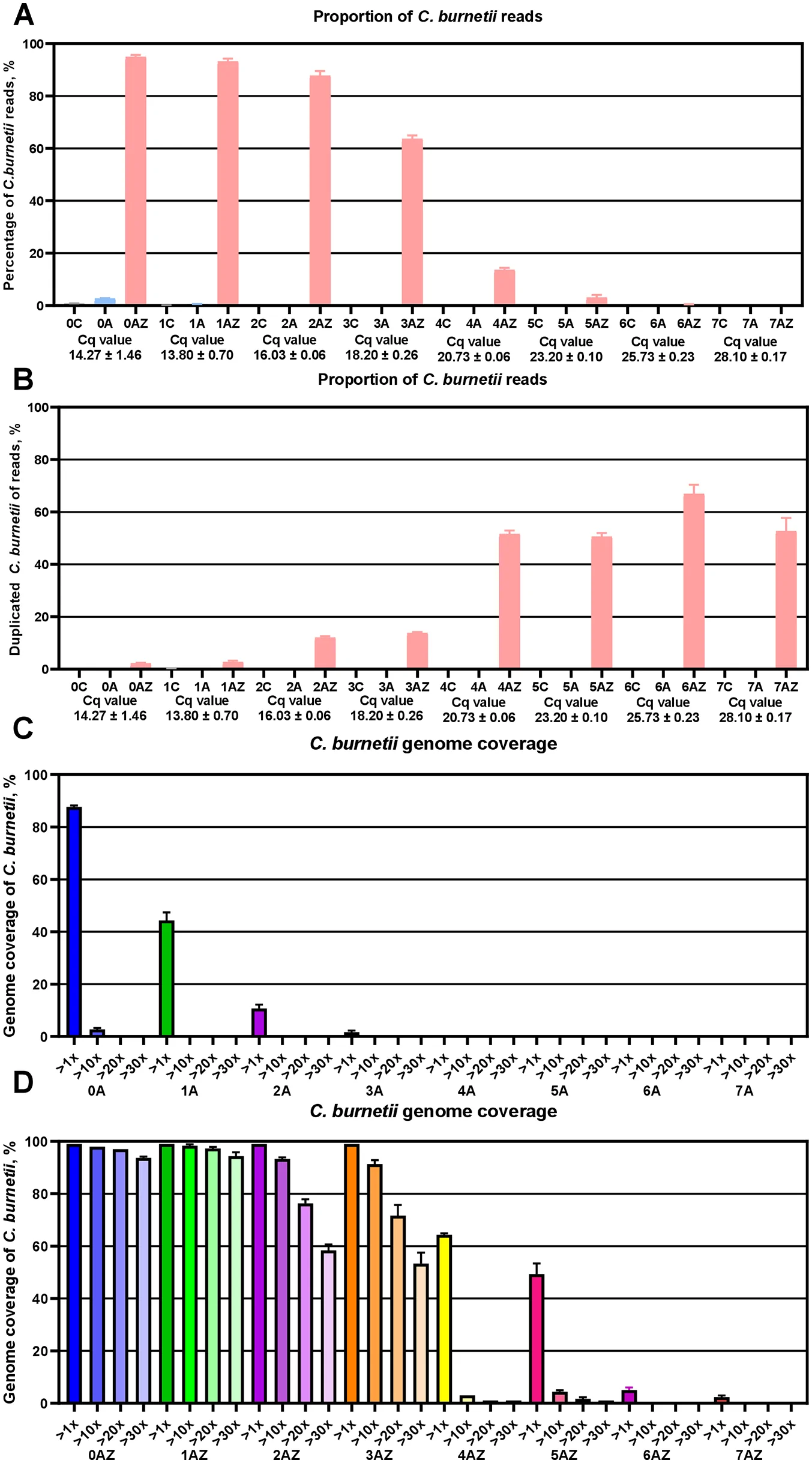
Evaluation of enrichment efficiency across a 5-fold dilution series of *C. burnetii* DNA. **(A)** Proportion of *C. burnetii* reads in C – non-enriched, A – SWGA-amplified, and AZ – SWGA + hybrid-captured libraries (mean ± SD, %). **(B)** PCR duplication rates in C – non-enriched, A – SWGA-amplified, and AZ – SWGA + hybrid-captured libraries (mean ± SD, %). **(C)** Genomic coverage of SWGA-amplified samples at 1 × , 10 × , 20 × , and 30× (mean ± SD, %). **(D)** Genomic coverage of SWGA + hybrid-captured samples at 1 × , 10 × , 20 × , and 30× (mean ± SD, %).

Collectively, these data demonstrate that the sequential two-stage enrichment method works effectively up to approximately dilution 3, with performance declining notably at dilutions 4 and beyond (Fig 2). The graphical visualization highlights the substantial improvement achieved through hybridization capture and the sharp decline in sequencing performance after dilution 4.

### Selective whole genome amplification

For this study, total DNA extracts from six sheep wool samples testing positive for *C. burnetii* by qPCR were selected. According to the manufacturer’s instructions for the AmpliSens *Coxiella burnetii* FL assay (InterlabService, Russia), Cq values in the range of 14–15 cycles reliably indicate the presence of the pathogen’s DNA. Aliquots containing 1.6–2.4 ng of total DNA from each sample were subjected to SWGA. This amplification step was critical, as the initial DNA amount was insufficient for standard library preparation, which typically requires higher input quantities. SWGA increased the total DNA yield by a factor of 1,188–2,344 (Table 6), providing sufficient material for downstream applications. The amplified material was then used to prepare NGS libraries for two purposes: (i) an interim assessment of the *C. burnetii* DNA fraction in the amplified samples and (ii) subsequent targeted enrichment.

**Table 6:** Total DNA yield after SWGA.

№	Sample ID	Cycle threshold before SWGA, Cq	Input DNA, ng	Output DNA, ng	Amplification Ratio
1	Q7533A	14.1	1.6	3600	2250
2	Q7537A	15.5	2.4	3300	1375
3	Q7543A	15.4	1.6	3300	2063
4	Q7544A	14.6	1.6	3300	2063
5	Q7545A	15.3	2.4	2850	1188
6	Q7546A	14.5	1.6	3750	2344

Following sequencing, we estimated the proportion of *C. burnetii* DNA in each SWGA-enriched library by aligning reads to the reference genome. The fraction of *C. burnetii* DNA ranged from 0.2% to 4.7%, with four of the six samples containing less than 1% of the target pathogen DNA (Table 7). PCR duplication rates were uniformly low across all samples, ranging from 0.52% to 0.98%. At a minimum 1 × read depth, the genomic coverage of the *C. burnetii* reference genome varied from 20% to 96%.

**Table 7:** Genomic coverage after SWGA.

№	Sample ID	Total reads. n	Reads aligned to the *C. burnetii* genome, n (%)	*C. burnetii* duplicated reads, n (%)	1x genomic coverage, %	10x genomic coverage, %	20x genomic coverage, %	30x genomic coverage,%
1	Q7533A	3321896	156310 (4.71)	1531 (0.98)	96	41	11	4
2	Q7537A	3237562	6314 (0.20)	52 (0.82)	20	0	0	0
3	Q7543A	3428386	21279 (0.62)	127 (0.60)	46	3	0	0
4	Q7544A	3241682	67031 (2.07)	417 (0.62)	86	11	2	1
5	Q7545A	3044036	16012 (0.53)	84 (0.52)	47	1	0	0
6	Q7546A	3626928	33200 (0.92)	204 (0.61)	61	4	1	0

### In-solution hybridization

DNA libraries were pooled in proportion to the previously determined *C. burnetii* DNA fractions in each sample and subjected to solution-based hybridization capture using in-house-generated biotinylated probes. The combined application of SWGA and hybridization capture markedly enhanced the sequencing efficiency for *C. burnetii*. Following enrichment, the proportion of sequencing reads aligning to the *C. burnetii* reference genome increased substantially across all six samples, reaching 83–95%, with PCR duplication rates ranging from 12.7% to 21.1% (Table 8). In two of the six samples, > 90% of the reference genome was covered at a depth exceeding 30 × . This integrated approach yielded five near-complete *C. burnetii* genomes (completeness up to 100%) and one partial genome assembly (Q7537AZ, 46.6% completeness), with estimated contamination levels below 2% for all assemblies. All WGS parameters are provided in Table 9.

**Table 8:** Read depth after Phi29 amplification and probe hybridization.

№	Sample ID	Total reads. n	*C. burnetii* mapped reads, n	Reads aligned to the *C. burnetii* genome, n (%)	*C. burnetii* duplicated reads, n (%)	1x genomic coverage, %	10x genomic coverage, %	20x genomic coverage, %	30x genomic coverage, %
1	Q7533AZ	5506378	5200764	5200764 (94.4)	660718 (12.7)	98	98	97	97
2	Q7537AZ	2058830	1709066	1709066 (83.0)	360279 (21.1)	76	49	40	34
3	Q7543AZ	2516316	2149286	2149286 (85.4)	436685 (20.3)	96	79	68	59
4	Q7544AZ	3621884	3244663	3244663 (89.6)	591172 (18.2)	98	97	94	90
5	Q7545AZ	1278218	1082948	1082948 (84.7)	155428 (14.4)	96	79	62	51
6	Q7546AZ	4008068	3542923	3542923 (88.4)	718137 (20.3)	97	90	83	77

**Table 9:** *De novo* genomic assembly metrics.

	Q7533AZ	Q7537AZ	Q7543AZ	Q7544AZ	Q7545AZ	Q7546AZ
Completeness (%)	100	46.646	82.735	99.9	77.4	93.27
Contamination (%)	0.099	0.199	0	0.699	1.729	1.796
Genome size (bp)	2070721	1099043	1944568	2110954	1777781	2116612
Contigs	140	485	1235	600	577	777
N50 (bp)	48343	9602	7636	18140	12447	14340
Longest contig (bp)	132663	35422	36922	75026	37843	44215
GC (%)	42.624	43.138	43.151	42.838	42.785	42.973
Genes	2131	1365	2866	2592	2219	2698

Nevertheless, considerable variability in sequencing yield was observed among the pooled samples, resulting in uneven genomic coverage and preventing uniform depth across all reconstructed genomes.

### Sequencing saturation analysis

**Fig 3:**
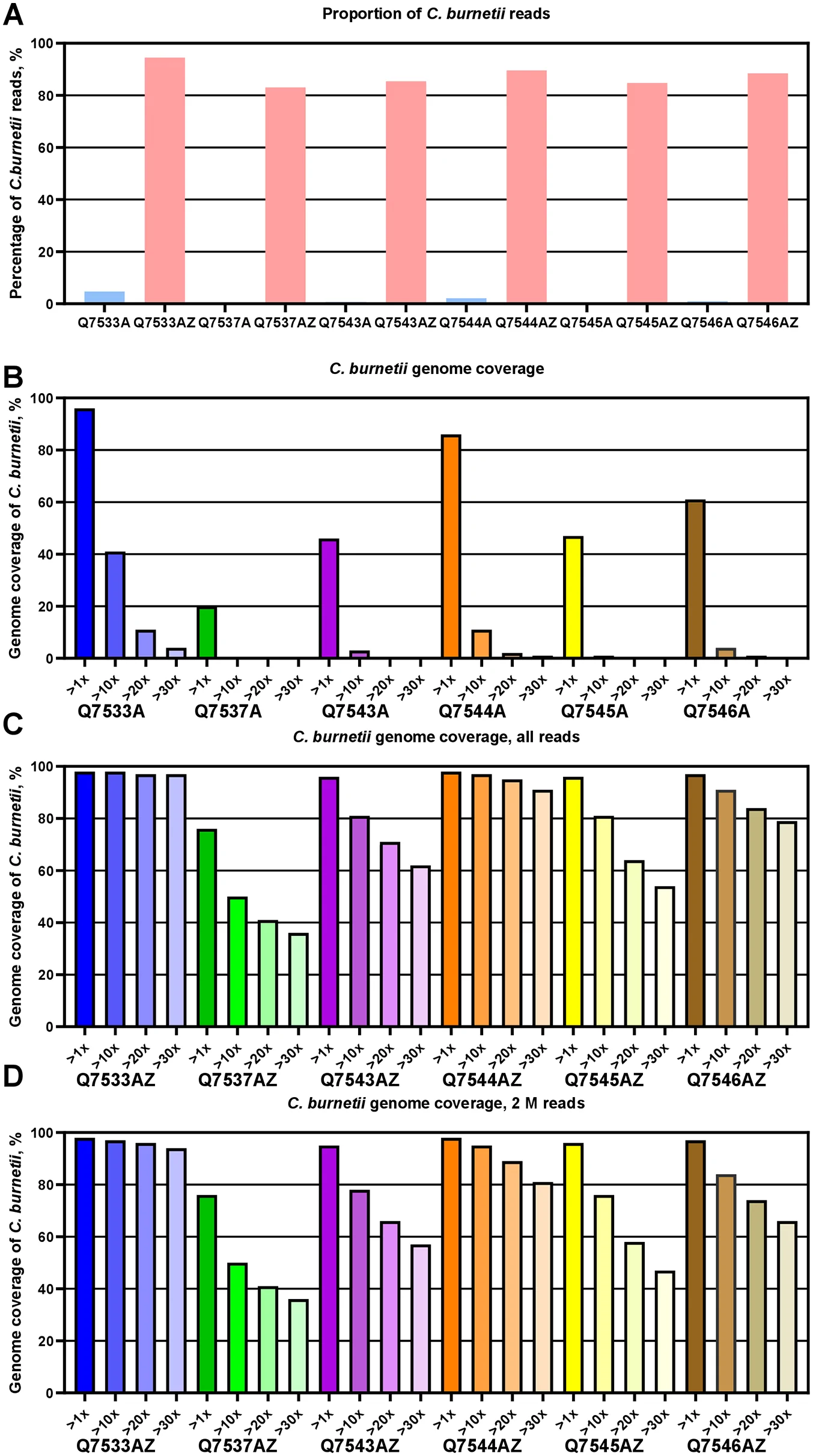
Sequencing results of *C. burnetii.* **(A)** Proportion of *C. burnetii* reads in sequenced samples following amplification. **(B)** Genomic coverage of samples after amplification. **(C)** Genomic coverage of samples after amplification and hybridization. **(D)** Genomic coverage profiles following amplification and hybridization. Sequencing depth was normalized to 2 million reads per sample, with the exception of sample Q7545AZ, which contained fewer reads.

**Fig 4:**
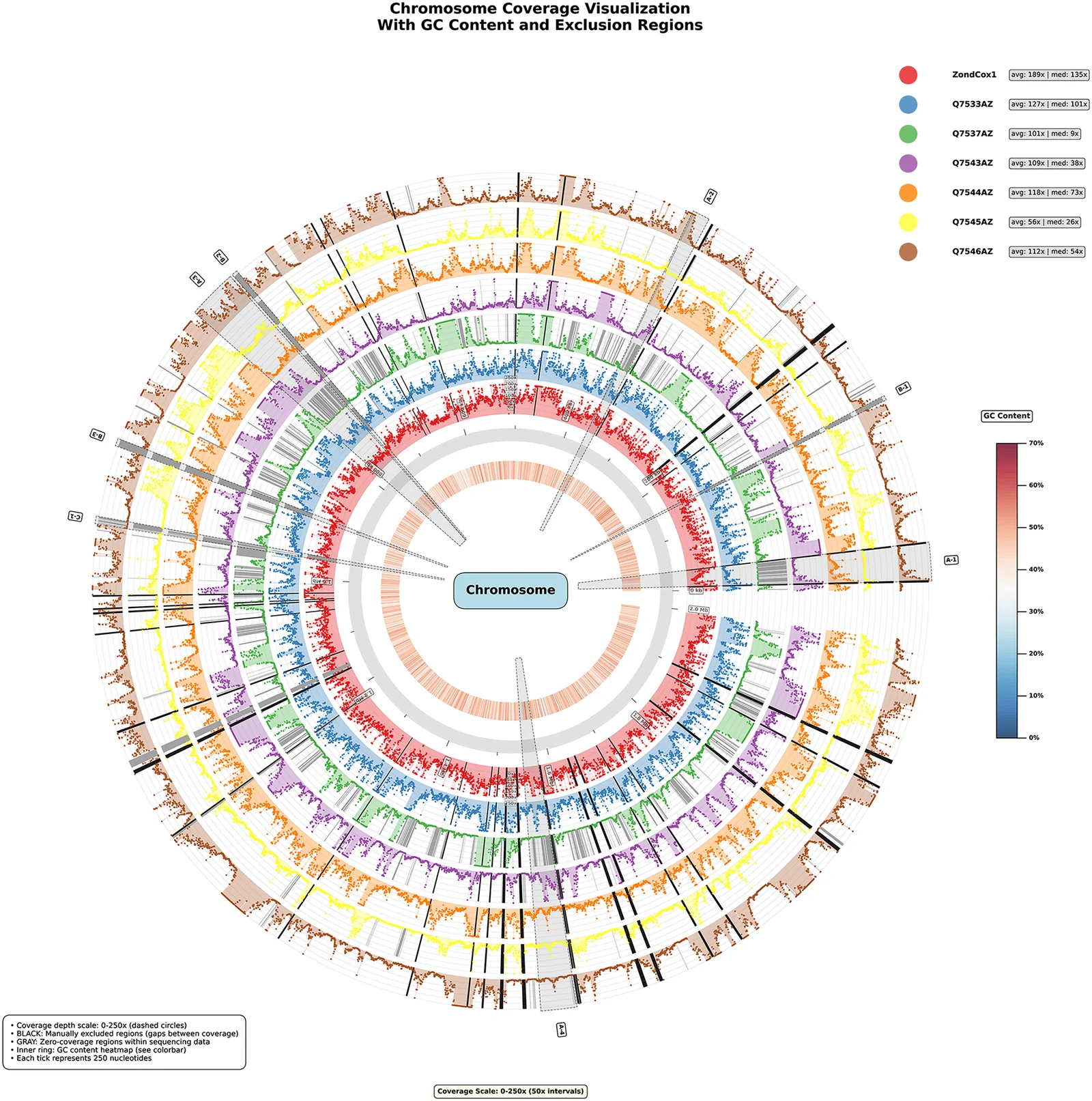
Visualization of *C. burnetii* genomic coverage density. The diagram shows coverage of the *C. burnetii* reference chromosome for the six sequenced samples and probes in non overlapping 250 bp windows. The diagram highlights three types of regions: (A1-A4) regions with variable coverage across all analyzed samples; (B1-B3) regions absent in all sequenced samples; and (C1) regions absent in the probe, but present in the analyzed samples.

We evaluated the reference genome coverage for the samples using incremental subsamples of reads. Sequencing saturation analysis indicated that the required read depth for complete *C. burnetii* genomic assembly varied significantly across samples, despite normalization of the samples by *C. burnetii* DNA content prior to pooling for hybridization (Table 10, Fig 3). Asymptotic coverage (≥1×) was attained at 2 million reads for samples Q7533AZ, Q7544AZ, and Q7546AZ. Sample Q7545AZ followed a similar curve, but was sequenced below this critical threshold. Sample Q7543AZ did not reach saturation, and sample Q7537AZ displayed a highly fragmented coverage profile with substantial zero-coverage regions (Fig 4). The complete result of *C. burnetii* DNA enrichment is shown in Fig 3.

**Table 10:** Sequencing saturation analysis.

Sample ID	№	Total number of reads, n	*C. burnetii* mapped reads, n	*C. burnetii* mapped reads, *%*	1x genomic coverage, %	10x genomic coverage, %	20x genomic coverage, %	30x genomic coverage, %
Q7533AZ	1	500000	472161	94.4	98	89	64	41
	2	1000000	944273		98	96	89	77
	3	1500000	1416588		98	97	95	89
	4	2000000	1888868		98	97	96	93
	5	2500000	2361164		98	97	97	95
	6	3000000	2833389		98	97	97	96
	7	3500000	3305817		98	97	97	97
	8	4000000	3778097		98	97	97	97
Q7537AZ	1	500000	415694	83.0	63	32	24	19
	2	1000000	830017		70	41	31	26
	3	1500000	1244794		73	45	36	31
	4	2000000	1660027		76	48	39	34
Q7543AZ	1	500000	426851	85.4	89	49	30	21
	2	1000000	853569		93	65	48	36
	3	1500000	1281556		95	72	58	47
	4	2000000	1708682		95	76	64	54
	5	2500000	2135347		96	79	68	59
Q7544AZ	1	500000	447919	89.6	97	73	47	31
	2	1000000	895908		97	88	72	57
	3	1500000	1343859		97	93	82	71
	4	2000000	1791281		98	95	88	79
	5	2500000	2239348		98	96	91	84
	6	3000000	2687279		98	96	93	87
	7	3500000	3135291		98	97	94	90
Q7545AZ	1	500000	423182	84.7	94	58	37	26
	2	1000000	847108		96	74	56	44
Q7546AZ	1	500000	442183	88.4	93	59	39	26
	2	1000000	884068		95	74	58	46
	3	1500000	1326618		96	80	67	57
	4	2000000	1768005		97	84	73	64
	5	2500000	2210046		97	86	76	69
	6	3000000	2651650		97	88	79	72
	7	3500000	3093595		97	89	81	75
	8	4000000	3535762		97	90	83	77

### Phylogenetic analysis

**Fig 5:**
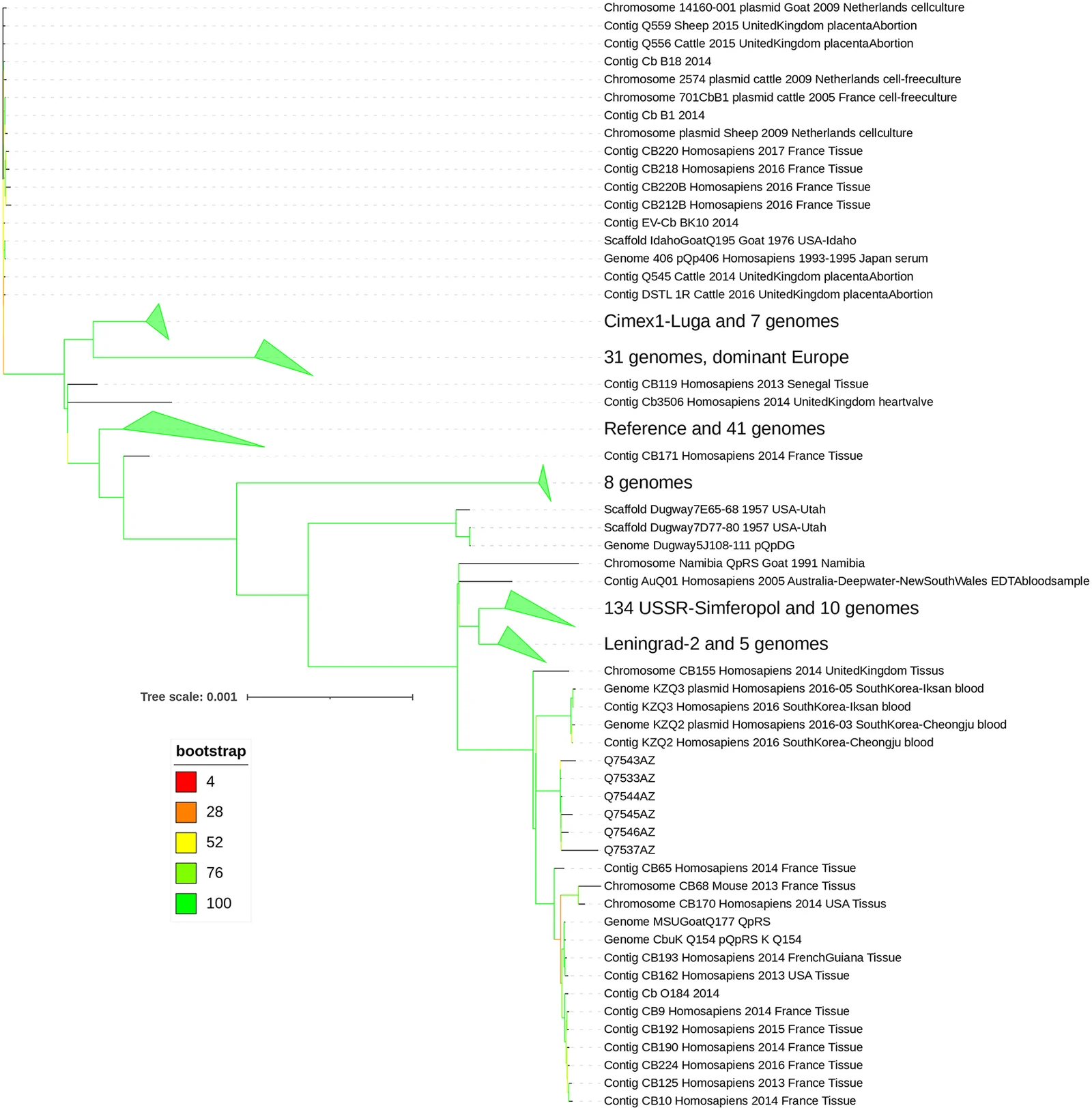
Phylogenetic analysis of *Coxiella* genomes obtained in this study.

The *de novo* assemblies of *C. burnetii* genomes generated in this study were subjected to phylogenetic analysis, with the reference genome RSA 493 (GCF_000007765.2) serving as the outgroup. The analysis included all publicly available *C. burnetii* genomic assemblies from GenBank and RefSeq databases retrieved at the time of the study. A condensed phylogenetic tree is presented in Fig 5, while the full phylogenetic tree, including all analyzed genomes, is provided in S1 Fig.

The tree was constructed using the maximum likelihood method based on *Coxiella* genomes retrieved from NCBI and those sequenced in this work.

As shown in Fig 5, the samples sequenced in this study cluster together in a single, exclusive group. This group is phylogenetically situated between a clade of South Korean strains and a clade largely consisting of European strains. However, our samples are separated by notable branch lengths from these neighboring groups, indicating genetic divergence. Other major branches in the tree are collapsed for simplicity and labeled broadly, with some labels encompassing additional strains from Russia.

## Discussion

### Result of amplification and hybridization

The ability to sequence *C. burnetii* genomes directly from DNA preparations of biological material is of great importance for epidemiological studies within Q fever surveillance, given the difficulties in culturing the causative agent. However, the low proportion of *C. burnetii* DNA in such preparations, along with the small total amount of DNA that can be isolated from samples like animal wool, dust, etc., necessitates the use of preliminary amplification and enrichment methods.

A method for specific enrichment of DNA preparations using Phi29 DNA polymerase was previously described [28], demonstrating efficacy on DNA from goat vaginal swabs. We applied this method to DNA preparations from sheep wool samples in which *C. burnetii* DNA had previously been detected during routine surveillance. Amplification for 12 hours increased the total DNA amount by 1,200–2,300-fold, providing sufficient DNA for subsequent experiments (Table 6).

Subsequent NGS and bioinformatic evaluation revealed that the proportion of reads aligning to the *C. burnetii* reference genome after SWGA ranged from 0.20% to 4.706%. In the most favorable case (sample Q7533A, ~ 3.3 million total reads), this translated into 96% coverage of the reference genome (Table 7). However, in samples with lower pathogen DNA fractions, genomic coverage remained below 1%. Due to limited material, we were unable to sequence the original, non-amplified DNA extracts to determine the baseline pathogen fraction. Nevertheless, based on Cocking *et al*., SWGA typically provides 68–147-fold enrichment of bacterial sequences [28]. While valuable, this level of enrichment is often insufficient for reliable genomic assembly or genotyping without prohibitively deep (and costly) sequencing.

To further enhance target recovery, we implemented an additional enrichment step: solution-based hybridization capture using custom dsDNA probes. Literature reports describe various probe-generation strategies based on genomic DNA or amplicon pools, successfully applied to pathogen sequencing [33], mitochondrial DNA capture [34], and ancient human DNA studies [35]. When genomic DNA of the target organism is available, probe synthesis is straightforward, rapid, and easily customizable, for instance, by combining DNA from multiple strains or species to broaden capture specificity.

Critical steps in probe preparation include DNA fragmentation and incorporation of biotin labels for streptavidin-based pull-down. Current protocols employ either physical or enzymatic fragmentation, with the choice often reflecting laboratory preferences. Biotinylation can be achieved by ligating biotinylated adapters to fragmented DNA [34,35] or enzymatically via terminal transferase-mediated addition of biotinylated nucleotides [33]. However, both approaches rely on enzymatic reactions (ligation or tailing) whose efficiency is difficult to quantify and require specialized reagents (e.g., labeled oligonucleotides or additional enzymes), thereby complicating workflow and increasing costs.

Given our limited supply of *C. burnetii* DNA, we sought a streamlined, cost-effective probe synthesis method that minimized the need for additional reagents. We therefore performed WGA using Phi29 polymerase in the presence of biotin-15-dCTP, followed by enzymatic fragmentation using components from a standard library preparation kit (NadPrep EZ DNA Library Prep Kit v2). Aside from the biotinylated nucleotide, all required reagents were already in use for other steps of the workflow. This strategy enabled efficient generation of sufficient probe quantities from minimal starting material.

With the dNTP:biotin-15-dCTP ratio used, approximately one in every 25 cytosines in the amplified DNA was biotinylated. Given an average probe length of ~300 bp, this ensured at least one biotin moiety per molecule, even in AT-rich regions, which is sufficient for efficient capture. This approach with WGA for the whole genome hybridization probe synthesis is virtually universal and potentially may be applied to any genomic DNA sample, including the DNA from bacterial or viral strains, animals, plants, etc.

An additional characteristic of our probes was the presence of chicken DNA contamination (~35%) insofar as the *C. burnetii* M44 strain used for probe generation was propagated in chicken embryos. Prior to use, we assessed probe composition via NGS, and *C. burnetii* DNA accounted for approximately 46% of the total (Table 1). In our case, this did not compromise the specificity of enrichment. Following hybridization, *Coxiella* DNA constituted 83–95% of the sequencing libraries. This value is comparable to, or even exceeds, that typically achieved with commercial target enrichment panels, such as human exome or mitochondrial baits [36,37]. This finding supports the feasibility of using DNA from contaminated, or host-derived, sources of intracellular pathogens for probe design, provided the probes are applied to DNA samples originating from evolutionarily distant hosts.

Hybridization capture using these in-house probes dramatically improved target representation. The fraction of *C. burnetii* reads increased to 83–97%, and reference genome coverage rose to 87–95%, including even the samples where post-SWGA pathogen read fractions were below 1% (Tables 7,8). *De novo* assemblies achieved 46.6% to 100% completeness as assessed by CheckM2. Although target enrichment efficiency and sequencing depth varied across samples, as few as 2 million reads (equivalent to 1 million read pairs) can provide sufficient coverage to achieve ≥1 × depth across the entire *C. burnetii* genome (Table 10). Such a sequencing depth is readily achievable on most second-generation sequencing platforms, including Illumina’s MiSeq system.

Phylogenetically, all samples clustered into a single, well-supported clade (Fig 5), an expected pattern for isolates originating from the same region. This result serves as a key validation of our two-stage enrichment approach, confirming its ability to yield genomes from complex native samples that are directly applicable for resolving fine-scale transmission dynamics and population studies.

### Epidemiological significance

This study demonstrates the efficacy of our approach to direct-genome sequencing for *C. burnetii* obtained directly from sheep wool, a sample type of high epidemiological relevance. Sheep are a well-documented reservoir in Q fever transmission, with wool serving as a significant vehicle for pathogen spread [38,39]. Infection typically occurs during handling, shearing, or via inhalation of contaminated dust [40,41]. The risks are exacerbated by the fact that *C. burnetii* can remain viable in wool for 7–10 months at room temperature [42]. This persistence aligns with high observed contamination rates [40] and significant occupational risk, evidenced by seropositivity in over 50% of wool sorters in some studies [41]. The successful application of our WGS approach to this challenging sample material confirms its potential for detailed source attribution and outbreak investigation representing a methodological reserve that may be deployed in targeted epidemiological scenarios rather than routine surveillance. While its utility for wool from various sources appears promising, applicability to other environmental samples (e.g., dust, hay) requires further validation.

### Technical considerations, limitations, and opportunities for optimization

The combined use of SWGA and solution-based hybridization capture significantly enhances the efficiency of sequencing target genomes, such as that of *Coxiella burnetii,* directly from complex biological or environmental samples. This dual-enrichment strategy reduces the sequencing depth required for meaningful genomic analysis and thereby extends the feasibility of pathogen genomics to laboratories equipped only with low-throughput NGS platforms (e.g., Illumina MiSeq). However, this approach entails several technical caveats. The multi-step workflow includes multiple enzymatic amplification stages (Phi29-based SWGA followed by post-capture PCR), each of which can introduce biases and artifacts.

The initial SWGA step enables substantial multiplication of input DNA, making it possible to construct sequencing libraries from trace amounts of starting material while simultaneously enriching the target DNA fraction. Although Phi29 DNA polymerase is renowned for its high fidelity, the introduction of sporadic polymerase errors cannot be entirely ruled out. In scenarios where the amount of target DNA is extremely limited, approaching single genomic copies, such errors may become indistinguishable from genuine sequence variants, potentially confounding downstream analyses such as SNP calling or strain identification.

Another critical limitation associated with Phi29-based amplification is the highly uneven, “patchy” nature of genomic coverage it can produce when the input DNA concentrations are low. When DNA is scarce, stochastic effects dominate: some genomic regions may be omitted from the initial reaction entirely; others may become overrepresented (amplified from even a single template molecule). Consequently, coverage plots often exhibit sharp discontinuities, with large gaps adjacent to regions of extreme depth (Fig 4). While average genomic coverage may appear sufficient, this metric is misleading, as it obscures underlying fragmentation and the absence of biologically relevant segments. This uneven coverage poses a major obstacle to reliable *de novo* assembly and comprehensive genomic analysis.

The observed unevenness in genomic coverage cannot be attributed to the hybridization probes themselves. Indeed, the genomic regions missing from coverage differ between samples, suggesting a stochastic rather than systematic cause. It is likely that the *Coxiella burnetii*-derived DNA in the original samples represented only partial fragments of the genome, with certain regions entirely absent. Consequently, only those genomic segments physically present in the initial material could be amplified; regions that were not present could not be reconstructed. This is a classic “all-or-nothing” limitation characteristic of analyses involving highly degraded or ultra-low-input DNA. This represents a fundamental physical constraint, and the most reliable strategy to mitigate it is to increase the quantity of input DNA.

Furthermore, it is critical to recognize that errors introduced during the earliest cycles of amplification can be propagated and amplified alongside authentic sequences. Such artifacts may appear as high-confidence variants due to their high read depth, making them difficult to distinguish from true biological mutations without additional validation.

SWGA relies on the use of random primers designed to bind short DNA motifs that are statistically overrepresented in the target genome relative to background (e.g., host or environmental) DNA. These motifs were originally selected based on a reference *C. burnetii* genomic sequence. However, in divergent strains or lineages, genomic regions lacking these preferred motifs may fail to prime efficiently and thus be systematically underrepresented or entirely missed. This introduces a potential bias against genomic variants that differ significantly from the reference used for primer design. To minimize this risk, it is essential to periodically re-evaluate primer sets against newly deposited *C. burnetii* genomes in public databases (e.g., GenBank, RefSeq). Regular *in silico* assessment of primer binding site conservation across diverse strains will help ensure continued efficacy of the enrichment protocol and reduce the likelihood of missing lineage-specific genomic regions.

Another critical factor to consider is the double PCR amplification of sequencing libraries, which can introduce additional artifacts and biases into the final sequencing data. According to the protocol, an initial pre-capture PCR is performed with 12 amplification cycles, followed by a post-capture PCR with an additional 20 cycles. While such extensive amplification is often unavoidable when starting with extremely low DNA inputs, it carries two major drawbacks: (1) it exacerbates unevenness in genomic coverage due to preferential amplification of certain fragments; and (2) it increases the likelihood of polymerase-derived sequencing errors, especially if they occur early in the amplification process and are subsequently propagated across thousands of copies.

To mitigate these issues, the number of PCR cycles should be minimized wherever feasible. In particular, if the Phi29-based WGA yields sufficient DNA quantity and quality, the pre-capture PCR step may be reduced to the absolute minimum thereby preserving library complexity and reducing the accumulation of amplification artifacts. Similarly, post-capture PCR should be carefully titrated to the lowest number of cycles necessary to obtain adequate library yield for sequencing. Whenever possible, the use of high-fidelity polymerases and unique molecular identifiers (UMIs) can further help distinguish true biological variants from PCR- or sequencing-derived errors.

The hybridization step itself can also introduce artifacts, particularly when the probe design does not fully represent the genetic diversity of the target pathogen. In this study, we used in-house probes derived from the genomic DNA of a single *C. burnetii* reference strain (M44) propagated in chicken embryos. If this strain lacks certain regions present in environmental or clinical isolates, those regions in the samples may fail to hybridize efficiently and consequently remain unsequenced. To achieve more comprehensive and unbiased capture of diverse *C. burnetii* genomes, we plan to prepare future probe sets using a pooled mixture of genomic DNA from multiple, genetically distinct strains, thereby broadening genomic representation and improving capture uniformity across lineages.

Furthermore, to conserve reagents and reduce hands-on time, hybridization is often performed on pooled libraries. While this strategy is well justified in contexts such as human exome or gene-panel sequencing, where the proportion of target DNA is relatively consistent across samples and equal library pooling yields balanced post-capture representation, it is problematic in studies like ours, where the fraction of target DNA varies dramatically (by up to an order of magnitude or more) between samples and is initially unknown. In our pilot experiment, we mitigated this issue by first performing low-pass sequencing after Phi29 amplification to estimate the *C. burnetii* DNA fraction in each sample and then normalizing library pooling accordingly. This pre-normalization enabled relatively balanced sequencing yields across the six samples, although considerable variability in final data output still persisted.

However, such a two-step sequencing-and-normalization workflow is impractical for routine diagnostics or large-scale surveillance. Therefore, for applications involving highly variable or unknown pathogen loads, especially in environmental or veterinary samples, we recommend avoiding library pooling prior to hybridization. Instead, individual sample capture, followed by post-enrichment pooling, offers a more reliable route to uniform genomic coverage and robust downstream analysis.

The method was validated using artificial DNA mixtures and assessed on a small set of wool samples. While the preliminary results are promising, extensive validation is required to determine the full scope of applicability and inherent limitations of the approach. Furthermore, the methodology can be further refined, for instance by optimizing primer design, minimizing PCR cycles, implementing two-stage hybridization, or using multi-strain probe pools to broaden capture efficiency. Nevertheless, the current data suggest that the protocol holds promise for enabling WGS of fastidious, hard-to-culture pathogens directly from complex matrices. Based on our dilution series data, the method performs effectively up to approximately Cq 20 without further optimization, defining the practical detection limit under the conditions tested. These findings indicate that this approach could significantly expand the accessibility of pathogen genomics, particularly for laboratories lacking high-throughput sequencing infrastructure or the capacity to culture dangerous agents such as *C. burnetii*.

## Conclusion

In conclusion, we successfully implemented a two-step enrichment strategy to obtain WGS of *Coxiella burnetii* directly from native biological material without prior pathogen cultivation. To our knowledge, several aspects of our approach have not been previously reported in combination. First, we integrated two complementary enrichment methods: SWGA using Phi29 DNA polymerase, followed by solution-based hybridization capture. The initial SWGA step generated sufficient quantities of DNA with modestly increased target content, enabling library construction from trace inputs. The subsequent hybridization step dramatically enhanced target representation, achieving up to 95% *C. burnetii* reads in the final sequencing libraries.

Second, we employed custom dsDNA probes derived directly from cultured *C. burnetii* cells. A distinctive feature of our probe preparation was the use of WGA with Phi29 polymerase in the presence of biotinylated nucleotides. This is a simple and efficient method to generate abundant, uniformly labeled probes from limited starting material. Notably, because the bacterial strain was propagated in chicken embryos, the resulting probe pool contained a substantial fraction (~35%) of chicken DNA. Crucially, this host-derived contamination did not impair the efficiency of *C. burnetii* enrichment from complex samples (e.g., sheep wool), which themselves contained host DNA from an evolutionarily distant species. This finding demonstrates the feasibility of producing effective hybridization probes from intracellular pathogens grown in heterologous host systems (e.g., cell cultures or embryonated eggs) and applying them to samples derived from phylogenetically unrelated hosts.

Ultimately, this study establishes a robust proof-of-concept for recovering partial to near-complete genomes of *C. burnetii* directly from native biological specimens, offering a practical approach for genomic surveillance in laboratories lacking advanced culture facilities. While the current protocol is effective for samples with high pathogen loads, we recognize that extending this workflow to other difficult-to-culture agents, or to specimens with lower target abundance, will require pathogen-specific optimization and rigorous validation. Nevertheless, the integration of SWGA and custom hybridization capture provides a versatile methodological framework that, pending further refinement, could potentially be adapted to broaden the genomic surveillance of other fastidious intracellular pathogens.

## Supporting information

S1 Table All genomes used to construct the phylogenetic tree. (XLSX)

S2 Table Summary of sequencing data for the 5-fold dilution series (native, SWGA-amplified, and hybrid-captured libraries). (XLSX)

S1 Fig *Coxiella burnetii* phylogenetic tree, full version. Branch support values represent UFBoot percentages but are labeled as “bootstrap” on the figure for simplicity.
(TIFF)
